# Cobalt, nickel and zinc spinel ferrites with high transmittance and UV-blocking for advanced optical applications

**DOI:** 10.1038/s41598-025-99604-6

**Published:** 2025-05-13

**Authors:** Mai M. El-Masry, M. M. Arman

**Affiliations:** 1 Basic Science Dept., Higher Engineering Institute, Thebes Academy, Cairo, Egypt; 2 Materials Science Lab (1), Physics Department, Faculty of Science, Cairo University, Giza, Egypt

**Keywords:** CoFe_2_O_4_, NiFe_2_O_4_, And ZnFe_2_O_4_, Spinel ferrite, NIR and shortwave infrared sensing, Optical properties, Materials for optics, Materials science

## Abstract

This study successfully synthesized and characterized CoFe_2_O_4_, NiFe_2_O_4_, and ZnFe_2_O_4_ ferrite nanoparticles. The results showed that CoFe_2_O_4_ and NiFe_2_O_4_ exhibited ferrimagnetic behavior, while ZnFe_2_O_4_ demonstrated antiferromagnetic properties. These magnetic characteristics influence the material’s response to electromagnetic radiation, such as visible and infrared light. Optical studies revealed that CoFe_2_O_4_ had the highest radiation absorption, while ZnFe_2_O_4_ showed superior reflection and transmission. The ferrites’ band gap energies, ranging from 3.3 to 3.6 eV, played a key role in their optical properties, with higher energy absorption and lower energy reflection. The refractive index varied with photon energy, reaching its peak at lower energy levels due to oxygen vacancies. Additionally, the optical conductivity increased with higher photon energy, peaking at 4.3 eV. These findings suggest promising applications in light transmission and sensing, with ferrites offering versatile optical properties that can be tailored for various uses.

## Introduction

In this article, there are three different spinel ferrites, Co ferrite, Ni ferrite and Zn ferrite nano particles. Spinel ferrites (AB_2_O_4_) are a class of inorganic compounds. They are composed of divalent metal cations, A, and tetravalent metal cations, B, which are octahedrally coordinated and share oxygen ions to create a cubic close-packed structure. The physical and magnetic properties of these materials make them useful in various applications^1,2^.

The spinel ferrite has the same general mineral spinel crystal structure, in which iron (II) oxide, Fe_3_O_4_, serves as the prototype. In the spinel structure, each oxygen ion is shared by four metal cations, two of type A and two of type B. The metal cations are octahedrally coordinated, meaning that each cation is surrounded by six oxygen ions. Oxygen ions are arranged in a cubic close-packed structure, in which each oxygen ion is coordinated to eight metal cations. The spinel ferrites can be seen as a special case of the general ABX_3_ structure, in which the X site is occupied by oxygen and the A and B sites are alternately occupied by divalent and tetravalent metal cations^3–5^.

The magnetic properties of AB_2_O_4_ are determined by the cations on the A and B sites. If the A and B cations have the same magnetic moment, the material is non-magnetic. If the cations distributed in the A and B sites possess different magnetic moments, the material is magnetic. The magnetic moment of the spinel ferrites is calculated by the coupling between the A and B cations^4,6,7^.

Spinels ferrites, particularly those based on cobalt (Co), nickel (Ni), and zinc (Zn), have garnered significant attention due to their unique optical properties and potential applications in advanced optical devices. These materials exhibit high transmittance in the visible spectrum while effectively blocking ultraviolet (UV) radiation, making them ideal candidates for UV shielding, smart windows, and optoelectronic devices. The choice of this specific composition cobalt, nickel, and zinc spinel ferrites is driven by their tunable electronic structure, which arises from the synergistic effects of transition metal ions occupying tetrahedral and octahedral sites within the spinel lattice. This structural arrangement influences their bandgap energy, magnetic properties, and optical absorption characteristics, as highlighted in recent studies^8,9^. For instance, cobalt ferrites are known for their excellent magnetic properties and moderate optical transparency, while nickel ferrites offer enhanced stability and tunable UV-blocking capabilities^10^. Zinc ferrites contribute to improved transparency and reduced optical losses, as demonstrated in diamond-like carbon coatings and thin films^11^. Furthermore, the incorporation of these elements into a single-phase spinel structure allows for the optimization of both optical and magnetic functionalities, as evidenced by recent advancements in ceramic processing and material synthesis techniques^12^. By leveraging these advantages, the present study aims to systematically investigate the structural, optical, and functional properties of cobalt, nickel, and zinc spinel ferrites to pave the way for their integration into cutting-edge optical technologies.

In antiferromagnetic materials like ZnFe_2_O_4_, the magnetic moments tend to align in opposite directions, leading to a cancelation of the overall magnetic moment. This magnetic behavior can influence the absorption properties differently compared to ferrimagnetic materials. The magnetic ordering in ferrites can influence their electronic band structure. Changes in the band gap energy, as observed in ferrites, can affect the absorption and transmission of light. Variations in the band structure can lead to different energy levels and transitions, impacting the optical properties of the material.

Magnetic behavior is inherently linked to the spin of electrons. Spin-dependent processes, such as spin-dependent transitions and spin-flip scattering, can occur in magnetic materials. These processes can contribute to changes in the absorption and transmission properties of light, particularly in the presence of an external magnetic field. Magnetic behavior may introduce optical anisotropy in ferrites, meaning that the optical properties may vary with the direction of light propagation concerning the crystallographic axes. This anisotropy can lead to directional dependence in absorption and transmission properties.

Recent studies underscore the growing significance of spinel ferrites in optical technologies, leveraging their unique magnetic, electronic, and structural properties. The article^13^ highlights advancements in synthesis techniques for spinel ferrites, emphasizing their tunable bandgaps and absorption characteristics for photonic devices. Algarni et al. explores structural optimization of these materials, demonstrating enhanced light-matter interactions through controlled crystallite size and doping, critical for optoelectronic sensors^12^. Researchers elucidate the role of cation distribution and defects in modulating optical behavior, linking these features to applications in UV–Vis-NIR spectroscopy^11^. Researchers investigate nanocomposite spinel ferrites, revealing their potential in magneto-optical data storage and plasmonic systems^10^. A study in^9^ integrates spinel ferrites with diamond substrates, achieving robust optical coatings with high thermal stability for harsh-environment sensors. The interplay between magnetic ordering and optical responses was examined, advancing their use in spintronic-photonic hybrid devices^8^.

CoFe_2_O_4_, NiFe_2_O_4_, and ZnFe_2_O_4_ ferrite nanoparticles were investigated recently for their promising magnetic and optical properties. The ferrimagnetic behavior of CoFe_2_O_4_, coupled with its optical properties, can be utilized in magneto-optical devices. These devices exploit the interaction between magnetic and optical phenomena for applications like magneto-optical sensors and data storage^14–16^. A comparison between different properties of CoFe_2_O_4_, NiFe_2_O_4_, and ZnFe_2_O_4_ ferrite nanoparticles based on published work are listed in Table 1

**Table 1:** A comparison between different properties of CoFe_2_O_4_, NiFe_2_O_4_, and ZnFe_2_O_4_ ferrite nanoparticles.

Property	CoFe_2_O_4_	NiFe_2_O_4_	ZnFe_2_O_4_	Ref
Band Gap (eV)	1.0–1.5	2.0–3.5	1.5–2.2	^17–21^
Color	Black	Black/Gray	Yellowish/Brownish	
optical absorption	strong absorption in the visible and near-infrared regions	relatively lower optical absorption due to its wider band gap	moderate optical absorption	^17,21–23^
Photoluminescence	Weak	Weak	Visible/NIR	^24–27^
Magnetic Ordering	Ferromagnet	Weak Ferro/Ferrimagnet	Antiferromagnet	^18,19^
Curie/Neel Temp. (°C)	~ 590	~ 580	~ 600	^28–30^
Magnetic Anisotropy	Cubic	Cubic/Uniaxial	-	^31–33^
Response to External Stimuli	sensitive to temperature, pressure, and magnetic fields,	high stability and resistance to oxidation	sensitive to light, temperature, and pressure	^29,32–35^
Applications	photocatalysis and solar energy conversion	optoelectronic devices and transparent coatings	sensors, pigments, and photocatalysis	^18,19^

NiFe_2_O_4_'s magnetic and optical properties can be harnessed in magneto-optical modulators, where an external magnetic field modulates the intensity of light^36,37^. This can be useful in optical communication and signal processing. The magnetic behavior of NiFe_2_O_4_ nanoparticles, coupled with their optical properties, makes them suiTable for sensing applications. They can be utilized in magnetic field sensors or as components in optically based sensors for various environmental parameters^38,39^.

ZnFe_2_O_4_'s antiferromagnetic behavior and reflectance properties make it suiTable for optical filters^40^. These filters can selectively transmit or reflect specific wavelengths of light, finding applications in optical communication and imaging systems. ZnFe_2_O_4_ nanoparticles can be incorporated into transparent conductive films for applications in optoelectronic devices such as touchscreens, solar cells, and light-emitting diodes (LEDs). ZnFe_2_O_4_ may find application in LEDs due to its optical properties. Incorporating these nanoparticles into the LED structure can potentially enhance its performance and efficiency^41–43^.

The optical applications of CoFe_2_O_4_, NiFe_2_O_4_, and ZnFe_2_O_4_ ferrite nanoparticles span a wide range of technologies, from magneto-optical devices and sensors to photocatalysis and biomedical imaging. Their unique combination of magnetic and optical properties makes them versatile materials for advancing various fields of optics and photonics.

## Experimental work

### Synthesis of the nano ferrites

Cobalt nitrate, nickel nitrate, zinc nitrate, and iron nitrate, each with a purity of 99.9%, were bought from Sigma-Aldrich. The samples were synthesized by employing the citrate combustion method^44^. The stoichiometric blend of metal nitrates and citric acid, soluble in deionized water, was prepared with a citrate-to-nitrates ratio of 1:1. To achieve a neutral pH of 7, NH_4_OH solution was used for a pH change. The resulting solution underwent heating on a hot plate until ash formation occurred.

### Nanoferrites characterizations and analysis

The XRD technique was used to study the phase identification and determining the crystallite size via Bruker advance D8 diffractometer with a wavelength λ = 0.15418 mm. The diffraction peaks were indexed with the ICDD cards 22–1086, 44–1485 and 22–1012. FESEM was used to study the surface morphology of the samples was studied using FESEM Model Quanta 250 FEG. The function groups of the samples were studied using Fourier-transformed infrared (FTIR) which was scanned via a FTIR spectrometer (Nicolet iS10, USA, 1 cm^−1^ spectral resolution). The BET (Brunauer, Emmett, and Teller) surface area of the samples was studied using the BET, NLDFT Ads. model. The surface chemistry and electronic structure of the samples were studied using X-ray photoelectron spectroscopy (XPS, K-ALPHA, Thermo Fisher Scientific, USA). Cary 5000 UV–Vis–NIR Spectrophotometer (λ = 200–2000 nm, Version 1.12, double beam mode) was used to study the optical analysis of the samples. the magnetic properties of the ferrite were performed using the vibrating sample magnetometer (VSM; 9600–1 LDJ, USA).

## Results and discussion

### XRD

**Fig. 1:**
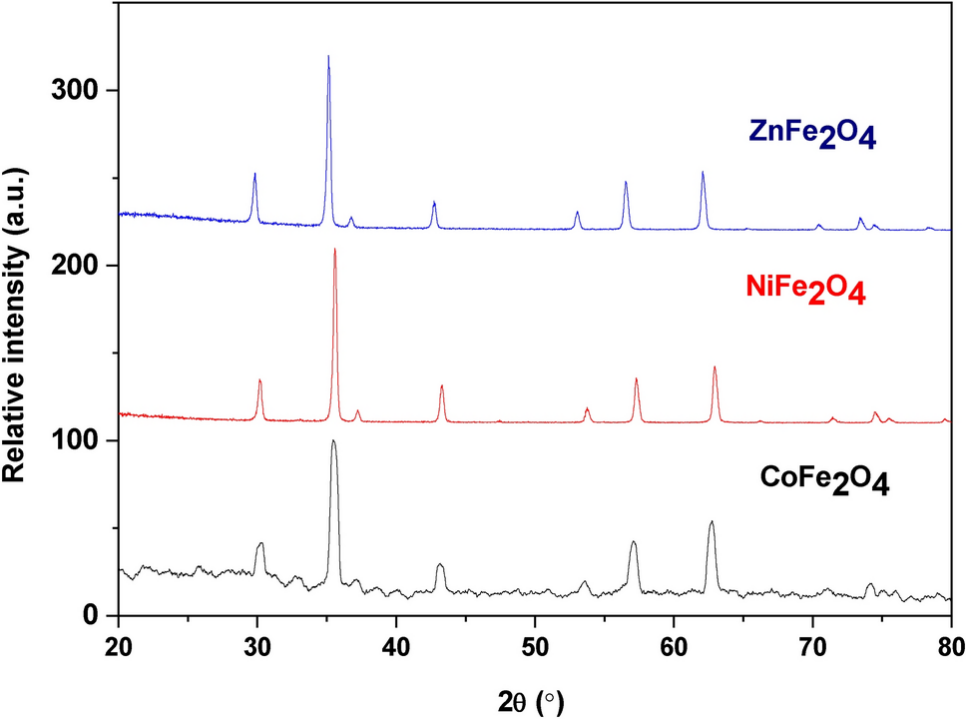
XRD Patterns of Nano Ferrite Samples. XRD patterns of CoFe_2_O_4_, NiFe_2_O_4_, and ZnFe_2_O_4_ nanoparticles, indexed with ICDD cards 22–1086, 44–1485, and 22–1012, confirming a single-phase cubic spinel structure. The calculated lattice parameters, unit cell volumes, and crystallite sizes validate the nano-scale dimensions of the samples.

Figure 1 shows the XRD pattern of the nano ferrite samples AFe_2_O_4_ (A: Co, Ni and Zn). The XRD data were indexed with the ICDD cards 22-1086, 44-1485 and 22-1012 for the CoFe_2_O_4_, NiFe_2_O_4_, and ZnFe_2_O_4_, respectively^45^. All samples crystallized in a single-phase cubic spinel structure (space group Fd-3 m) with no detectable impurities, confirming the phase purity of the synthesized ferrites. The sharp and well-defined diffraction peaks align with recent studies highlighting the importance of controlled synthesis conditions in achieving structurally ordered spinel ferrites^46^. the values of lattice parameter (a) for the samples were calculated on the bases on the cubic structure using the following equation^46,47^.

1 $$\frac{1}{{d}^{2}} =({h}^{2}+ {k}^{2}+ {l}^{2} ) \frac{1}{{a}^{2}}$$

where d denotes the d-spacing and (hkl) are the miller indices The unit cell volume (V) was derived from

2 $${\text{V}} = {\text{a}}^{{3}} ,$$

with values tabulated in Table 2). Notably, the lattice parameters and peak broadening trends correlate with cation distribution and crystallite size effects, as discussed in recent works on analogous spinel systems^48,49^.

**Table 2:** The lattice parameter (a), the unit cell volume (V), the theoretical density (D_x_) and the average crystalline size (L) for the samples.

Samples	a(Å)	V(Å^3^)	D_x_ (g/cm^3^)	L (nm) (XRD)
CoFe_2_O_4_	8.368	585.92	5.318	71.50
NiFe_2_O_4_	8.357	583.75	5.337	28.28
ZnFe_2_O_4_	8.426	598.22	5.324	30.26

The theoretical density (D_x_) of the samples was calculated using equation^46,49^ (3).

3 $$\text{Dx }= \frac{ZM}{{N}_{A}V}$$

where Z = 8, M denotes the molecular weight, N_A_ is the Avogadro’s number and V denotes the unit cell volume. Further validating the structural consistency of the synthesized ferrites. These findings are consistent with literature reports emphasizing the role of cation substitution (Co, Ni, Zn) in modulating spinel lattice dimensions while retaining structural integrity, a critical factor for optical and functional performance in advanced applications.

The Scherer equation^50,51^ was used to calculate the average crystallite size (L) of the spinel ferrites.

4 $$L = \frac{0.9 \lambda }{\beta cos\theta }$$

where λ refers the XRD wavelength, β denotes the full width at half maximum intensity and θ is the Bragg angle. The values of L are reported in Table 2 and confirm the prepared samples have nano dimensions^52^.

### FTIR

**Fig. 2:**
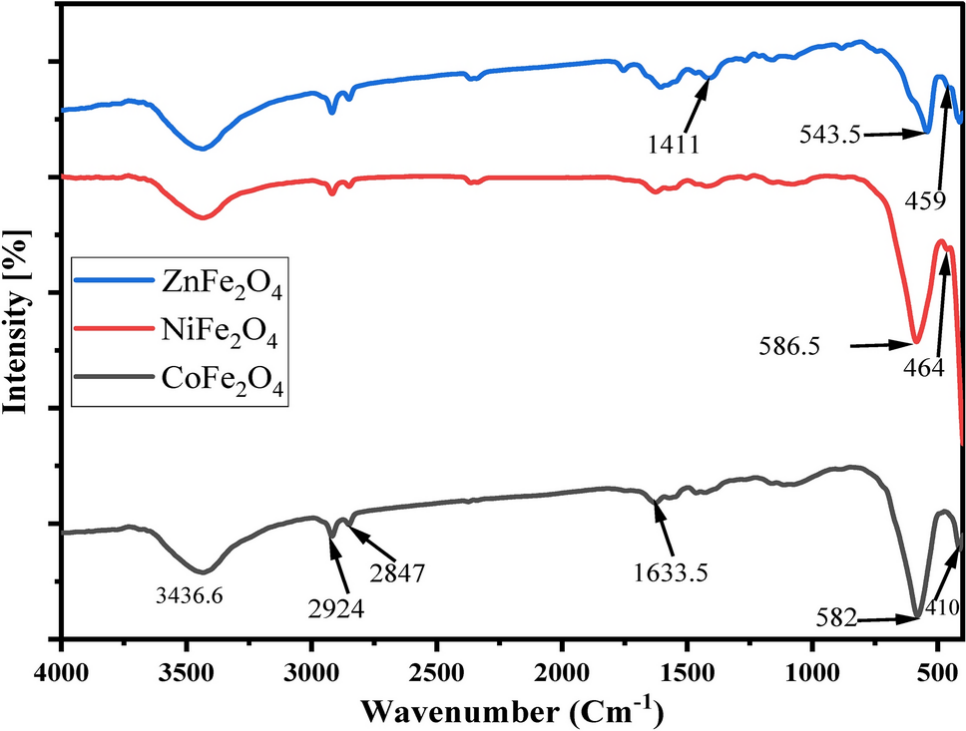
FTIR Spectra of Ferrite Samples. FTIR spectra of CoFe_2_O_4_, NiFe_2_O_4_, and ZnFe_2_O_4_ nanoparticles showing characteristic vibrational modes. Peaks include Fe–O and Co–O stretching (CoFe_2_O_4_), Ni–O and Fe–O modes (NiFe_2_O_4_), and Zn–O and Fe–O stretching (ZnFe_2_O_4_). Additional peaks represent functional groups such as C-H and C = O vibrations, indicative of the materials’ chemical composition and structural characteristics.

FTIR spectroscopy is a powerful tool for the characterization of inorganic materials like cobalt ferrite. This technique can provide information on the functional groups present in a sample, as well as the relative abundance of those groups. The infrared spectrum of nanoparticles is a valuable tool for understanding the structure and composition of this material. This information is important for optimizing the properties. Figure 2 illustrates the FTIR spectra of CoFe_2_O_4_, NiFe_2_O_4_ and ZnFe_2_O_4._

The infrared spectrum of cobalt ferrite nanoparticles is dominated by Fe–O and Co–O stretching vibrations, with some contribution from bending vibrations of the same groups. The symmetric and asymmetric stretching of the Fe–O bonds are well resolved, while the Co–O stretching mode is significantly broader.

The presence of these functional groups indicates that the cobalt ferrite nanoparticles are primarily made up of Co-Fe–O octahedra. This confirms what was previously predicted by X-ray diffraction studies of this material. The FTIR spectrum also provides quantitative information on the amount of each type of atom in the cobalt ferrite nanoparticles. From the relative intensities of the various stretching and bending vibrations, it is estimated that the cobalt ferrite nanoparticles contain approximately equal amounts of Fe and Co. The 3436 cm^-1^ peak is assigned to the C-H stretching vibration of aromatic species. The 2924 cm^-1^ peak is assigned to the alkene C-H stretching vibrational mode of olefins^53^. The 2847 cm^-1^ peak is attributed to the C-H bending region^54^. The 1633 cm^-1^ peak is attributed to the C = O stretching mode of carbonyl compounds. The 1411cm^-1^ peak in an FTIR spectrum is typically attributed to asymmetric stretching of carbon–carbon single bonds (stretching of C − C or C = C bonds). The 824 cm^-1^ peak is attributed to the C-N stretching vibration. Finally, the 582 cm^-1^ peak is assigned to the stretching vibration of the Fe–O-Fe bonded group, while the peak seen at 410 cm^-1^ is attributed to the stretching vibration of Fe–O bonds or iron oxyhydroxides and is known as the quaternary ferrite peak. It is a characteristic peak of the ferrite material, representing the mixed ferric-oxygen environment that is formed in the film^55–58^.

The FTIR spectrum of nickel ferrite was studied. The spectrum showed that the peak at 586 cm^-1^ corresponds to the Ni–O stretching mode, while the peak at 464 cm^-1^ corresponds to the Fe–O stretching mode. The results indicate that nickel ferrite has a symmetric structure, which agrees with previous studies.

The zinc ferrite FTIR spectrum was measured. A strong peak at 543 cm^-1^ refers to the Zinc-Oxygen (Zn–O) bond stretching frequency. A second peak at 459 cm^-1^ refers to the Ferric-Oxygen (Fe–O) bond stretching frequency. These peaks are expected for a zinc ferrite material, indicating the presence of both zinc and ferric oxide in the sample^55,56^.

### BET

**Fig. 3:**
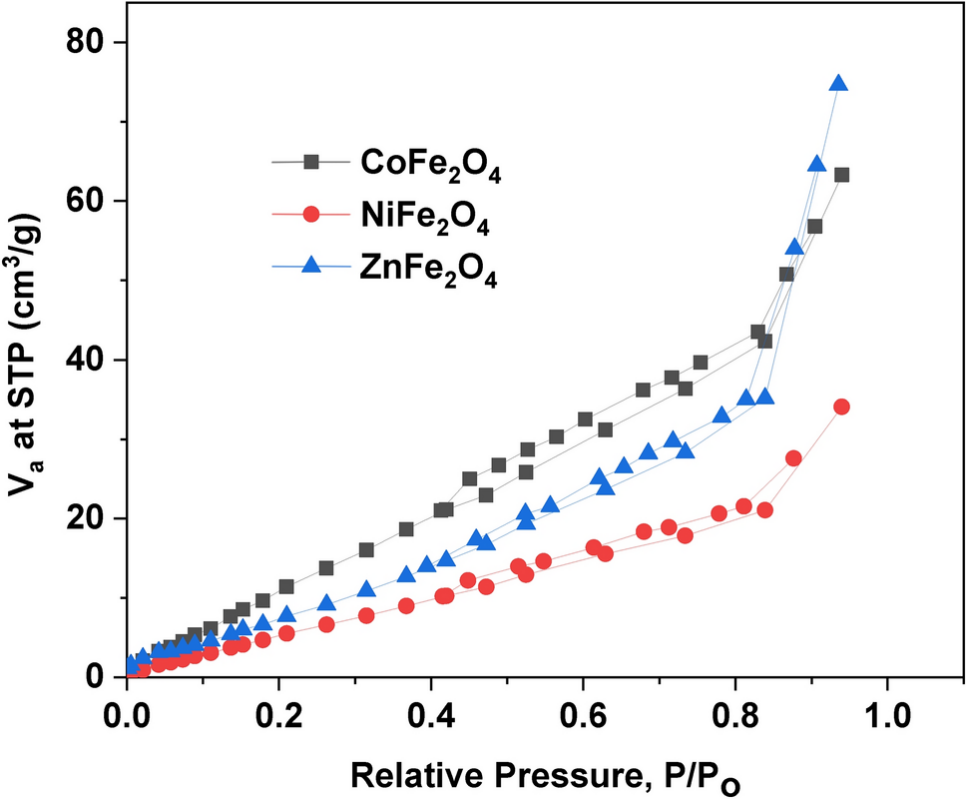
BET surface area of (CoFe_2_O_4_, NiFe_2_O_4_, and ZnFe_2_O_4_) samples.

Figure 3 shows the adsorption–desorption isotherm of CoFe_2_O_4_, NiFe_2_O_4_, and ZnFe_2_O_4_ nanoparticles. The BET isotherms of the samples are type II and H3 hysteresis loop according to IUPAC classifications^59,60^. Table 3 contains the values of total pore volume, specific surface area, and average pore diameter of the investigated ferrite samples^61,62^.

**Table 3:** The total pore volume, specific surface area, and average pore diameter of CoFe_2_O_4_, NiFe_2_O_4_, and ZnFe_2_O_4_ ferrites.

Samples	Specific surface area (m^2^/g)	Total pore volume (cm^3^/g)	Average pore diameter (nm)
CoFe_2_O_4_	120	0.098	3.26
NiFe_2_O_4_	44	0.053	4.72
ZnFe_2_O_4_	49	0.116	9.42

### FESEM

**Fig. 4:**
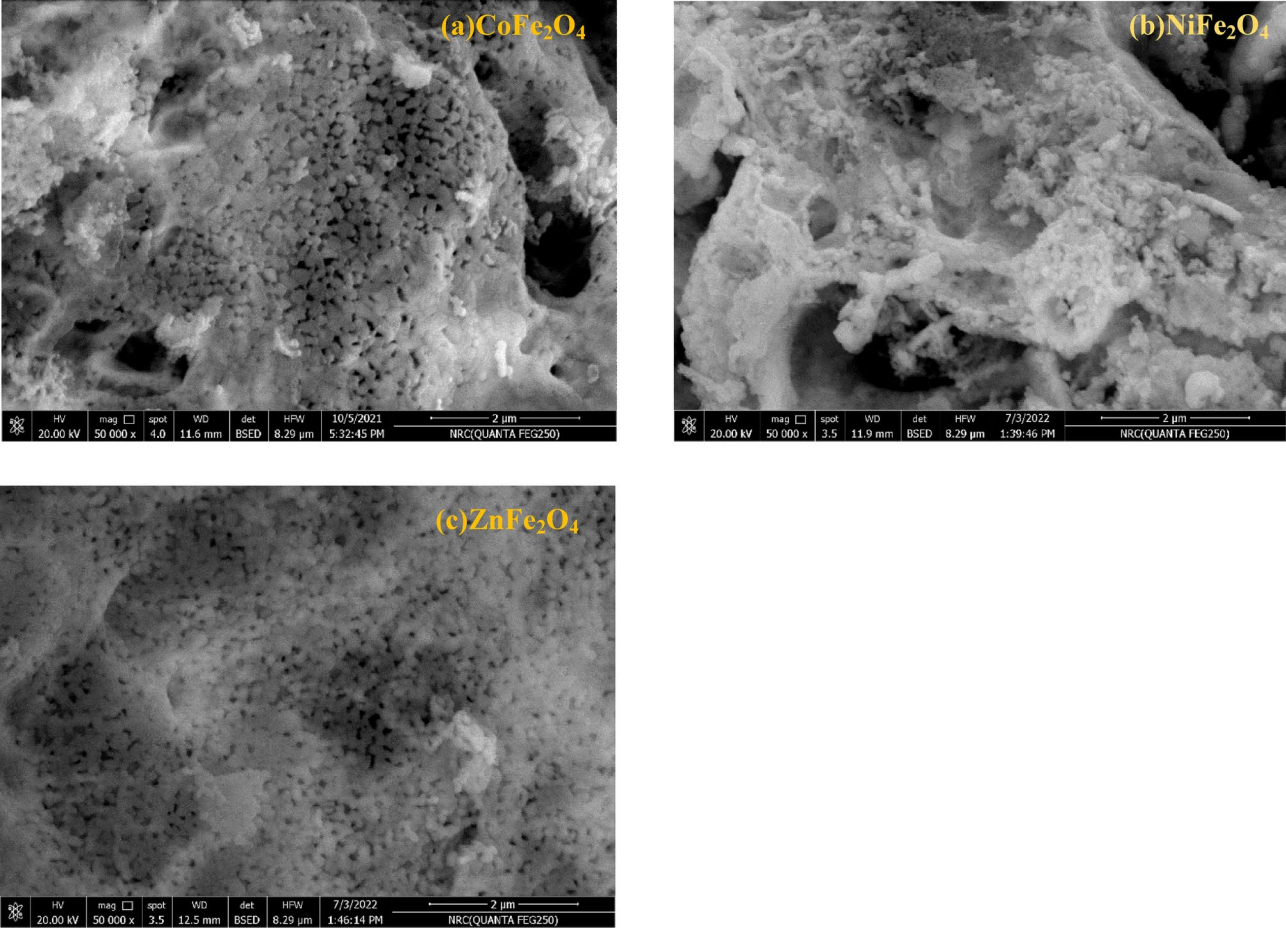
FESEM Images of Ferrite Morphology. FESEM images showing the morphology of CoFe_2_O_4_, NiFe_2_O_4_, and ZnFe_2_O_4_ nanoparticles. CoFe_2_O_4_ and ZnFe_2_O_4_ exhibit spherical clusters, while NiFe_2_O_4_ displays a cotton-like morphology. All samples confirm a porous structure with particle sizes in the nano range of 50–90 nm.

The morphology of AFe_2_O_4_ (A: Co, Ni and Zn) was studied using FESEM. The FESEM images confirm the presence of spherical nanoparticle clusters for CoFe_2_O_4_ and ZnFe_2_O_4_, while NiFe_2_O_4_ exhibits a unique cotton-like morphology, as shown in Fig. 4. These observations align with previous studies on spinel ferrites, where similar morphologies were reported to influence optical and magnetic properties^63,64^. The grain size of the CoFe_2_O_4_ and ZnFe_2_O_4_ range in 50–90 nm which assures that AFe_2_O_4_ (A: Co, Ni and Zn) were prepared in nano scale. Notably, the porous nature of the surfaces, as observed in the FESEM images, plays a critical role in enhancing the material’s functionality. Porous structures provide a higher surface-to-volume ratio, enabling increased interaction with incident light^63,65,66^. Porous structures provide a higher surface area, allowing for more interaction with incident light^66–68^. This characteristic is particularly advantageous for applications such as sensors, heavy metal removal, and optical filters, where enhanced light-matter interactions are desired^68^. Furthermore, the integration of porous ferrites into light-emitting devices can significantly improve their efficiency, making them suitable for advanced technologies like light-emitting diodes (LEDs) and laser systems.

### X- ray photoelectron spectroscopy (XPS):

**Fig. 5:**
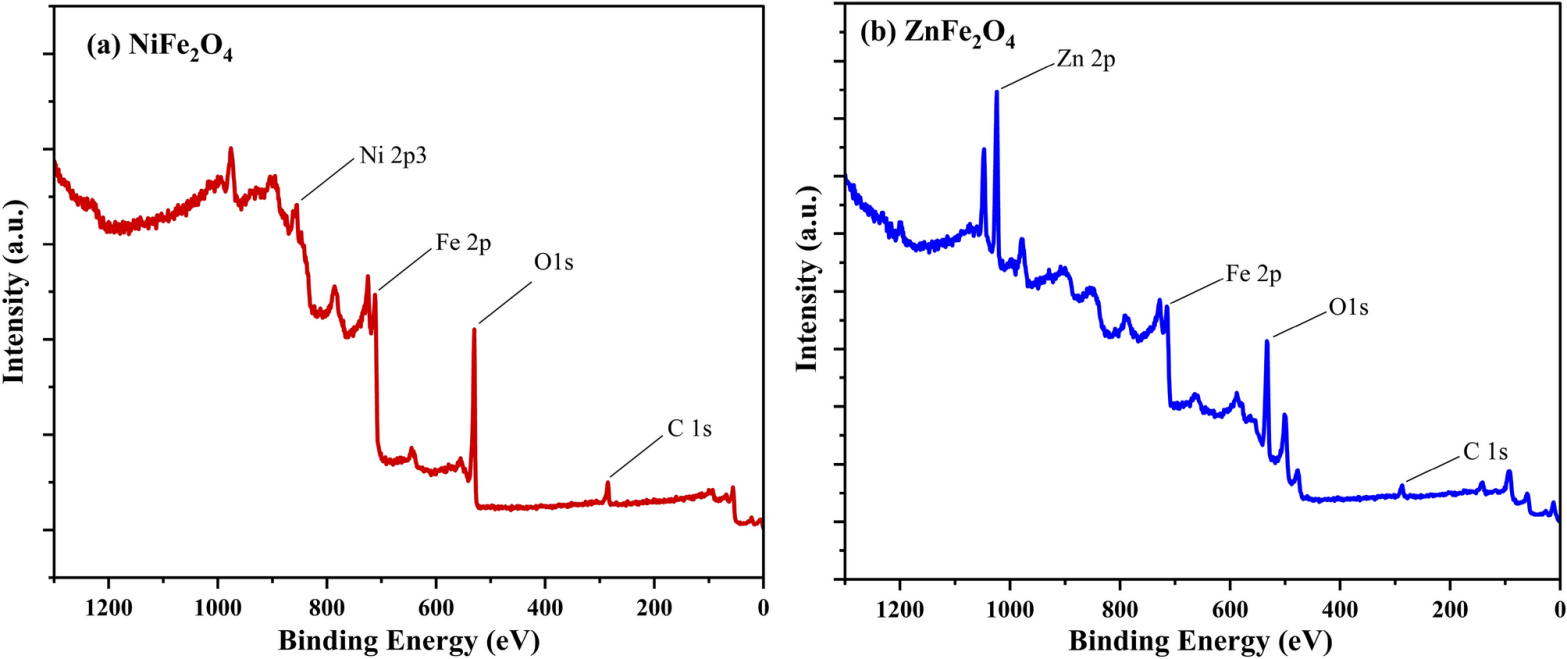
XPS spectra of (NiFe_2_O_4_ and ZnFe_2_O_4_).

X-ray photoelectron spectroscopy (XPS) is a critical tool for elucidating the surface chemistry, electronic structure, and cation distribution in spinel ferrites such as cobalt ferrite (CoFe_2_O_4_), nickel ferrite (NiFe_2_O_4_), and zinc ferrite (ZnFe_2_O_4_). In CoFe_2_O_4_, XPS spectra typically reveal Co 2p_3_/_2_ and Co 2p_1_/_2_ peaks at 781.57 eV and 796.56 eV, respectively, with satellite peaks characteristic of Co^2^_+_ in octahedral coordination, as published in our previous article^69^. As shown in Fig. 5 (a, b), the XPS spectra of NiFe_2_O_4_ and ZnFe_2_O_4_ exhibit distinct features^70^. The Fe 2p region displays binding energies of ~ 712 eV (Fe 2p_3_/_2_) and ~ 724 eV (Fe 2p_1_/_2_), confirming the presence of Fe^3^_+_ in both tetrahedral and octahedral sites, while the O 1s peak near 530 eV reflects metal–oxygen bonding. For NiFe_2_O_4_, the Ni 2p_3_/_2_ and Ni 2p_1_/_2_ peaks at 855 eV and ~ 873 eV, respectively, along with shake-up satellites, confirm Ni^2^_+_ in octahedral sites, with Fe^3^_+_ signatures consistent with those in CoFe_2_O_4_. In ZnFe_2_O_4_, the Zn 2p_3_/_2_ and Zn 2p_1_/_2_ peaks at ~ 1021 eV and ~ 1044 eV, respectively, indicate Zn^2^_+_ in tetrahedral sites, while the Fe^3^_+_ binding energies align with those in other ferrites. The O 1s spectra across all three materials consistently exhibit peaks near 530 eV, attributed to lattice oxygen. These XPS analyses provide valuable insights into cation valence states, site occupancy, and surface chemistry, which are pivotal for tailoring their magnetic, catalytic, and electronic properties for applications in spintronics, sensors, and energy storage. Table 4 highlights the cation distribution, oxidation states, and surface chemistry of both ferrites, with ZnFe_2_O_4_ showing carbon adsorption and NiFe_2_O_4_ exhibiting a cleaner surface.

**Table 4:** ZnFe_2_O_4_ and NiFe_2_O_4_ elemental compositions and chemical states.

ZnFe_2_O_4_	Peak BE	Atomic %	NiFe_2_O_4_	Peak BE	Atomic %
Zn2p	1022.32	18.22	O1s	530.31	57.08
O1s	530.91	49.45	Fe2p	712.03	28.88
Fe2p	712.48	16.36	C1s	285.33	0
C1s	285.48	15.96	Ni2p3	855.53	14.04

### Optical properties

**Fig. 6:**
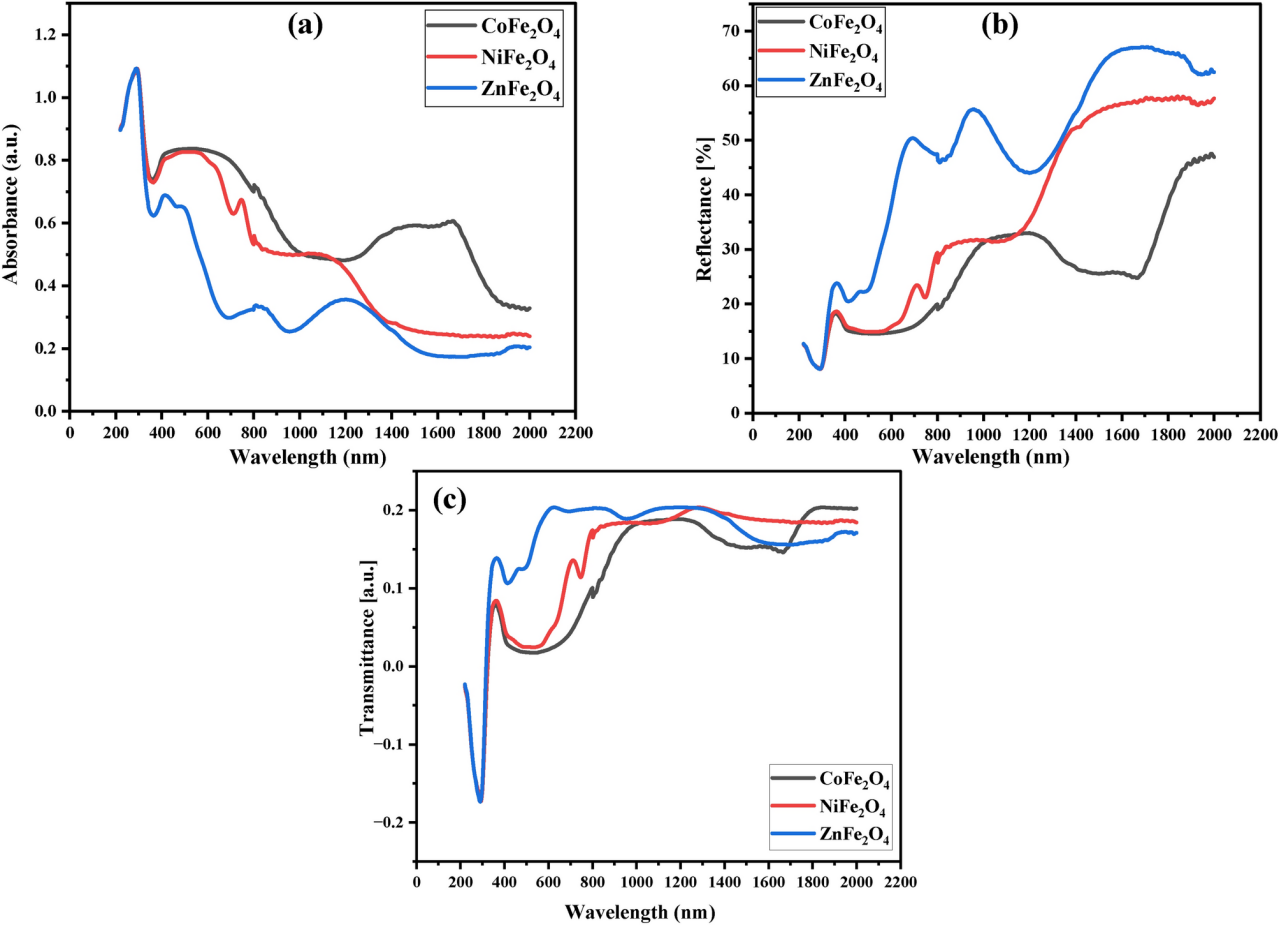
Optical Absorption, Reflection, and Transmission Spectra. Absorption (**a**), reflection (**b**), and transmission (**c**) spectra for CoFe_2_O_4_, NiFe_2_O_4_, and ZnFe_2_O_4_ nanoparticles across UV, visible, and NIR regions. CoFe_2_O_4_ demonstrates maximum absorption, while ZnFe_2_O_4_ exhibits the highest reflection and transmission, supporting diverse optical applications.

All the spinel ferrites under investigation absorbed radiation in the UV-region, while they reflected and transmitted radiation with high intensity in visible (VIS), near infrared (NIR) and short-wave infrared regions from 400 nm to 2 μm, as shown in Fig. 6 (a–c). The variation in optical band gap, Urbach energy, and optical absorption of these ferrites are caused by lattice distortions induced by different cations because of oxidation of A-site cations. These lattice distortions result in different energy levels and band gap widths. As a result, oxygen vacancies are produced in the structure which has a significant impact on the optical properties.

The prepared AFe_2_O_4_ (A: Co, Ni and Zn) ferrites could be used for blocking UV radiation, NIR (800–1100 nm) radiation, and shortwave infrared radiation sensors. The high transmission in the NIR region makes the samples investigated suiTable for solar energy collection. The ferrites exhibited excellent chemical stability in water, which enabled them to be used in various optical devices such as solid-state lasers, lasers, and optical fibers. The ferrites can also be used in optoelectronic components such as light-emitting diodes (LEDs) and photodetectors^71–76^.

**Fig. 7:**
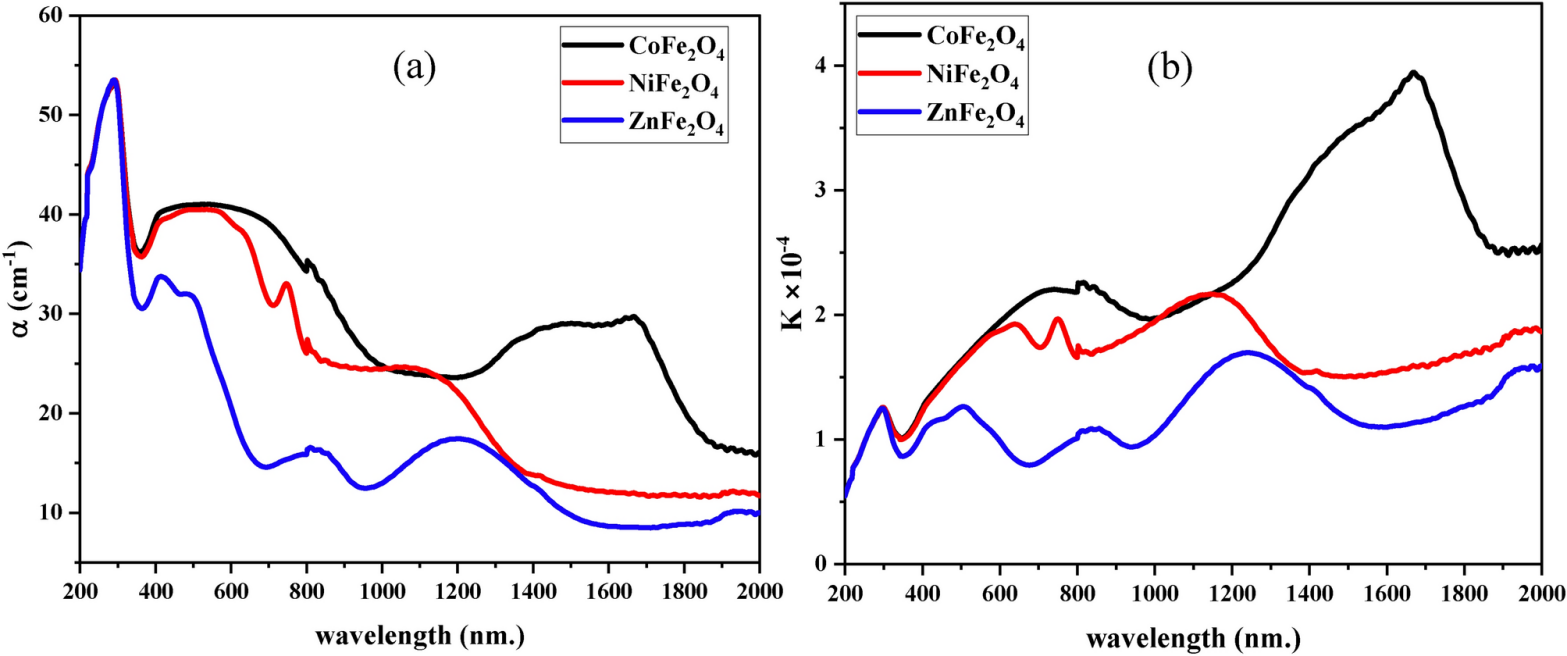
Absorption and Extinction Coefficients. Graphs of absorption (**a**) and extinction coefficients (**b**) versus wavelength for CoFe_2_O_4_, NiFe_2_O_4_, and ZnFe_2_O_4_ nanoparticles. CoFe_2_O_4_ shows the highest absorption efficiency, highlighting its potential for light-blocking applications.

CoFe_2_O_4_ was found to be the largest absorb of radiation among the studied ferrites ranging from 190 to 2000 nm, as shown in Fig. 6 (a). In contrast, Zn Fe_2_O_4_ was found to be the largest reflector and transmitter of radiation in the same range as shown in Fig. 6 (a, b). This suggests that these two ferrites are suiTable for different applications depending on their optical properties. By controlling their composition and fabrication conditions, the optical properties can be tailored to meet specific requirements (Fig. 7).

Using Tauc’s relation (Eq. 1) ^77^, we calculated the band gap energy (E_g_) values of CoFe_2_O_4_, NiFe_2_O_4_, and ZnFe_2_O_4_ ferrites to be ranging from 3.3 eV to 3.6 eV (listed in Table 5). These observed E_g_ values are consistent with the optical properties of the examined ferrites, as they absorb radiation at high incident photon energy and reflect radiation at lower energy of the incident photon. Moreover, the E_g_ values we obtained are in line with those reported in the literature.

5 $${\left(\boldsymbol{\alpha }{\varvec{h}}{\varvec{\nu}}\right)}^{{\varvec{x}}}={\varvec{B}}({\varvec{h}}{\varvec{\nu}}-\boldsymbol{ }{{\varvec{E}}}_{{\varvec{g}}})$$

where, B is a constant, ($h\nu )$ is the photon energy. x depends on the type of transition for x = 2, is direct allowed band gap **(Eg**_**dir.**_**)** and x = ½, **(Eg**_**ind.**_**)** indirect allowed band gap.

**Table 5:** direct band gap **(Eg**_**dir.**_**),** the indirect band gap **(Eg**_**ind.**_**)** and Urbach energy Eu of CoFe_2_O_4_, NiFe_2_O_4_ and ZnFe_2_O_4_.

Compound	Eg_Dir._ (eV)	Eg_ind._ (eV)	Eu_._ (eV)
CoFe_2_O_4_	3.3	1.9	0.14
NiFe_2_O_4_	3.5	2.1	0.30
ZnFe_2_O_4_	3.6	2.5	0.27

**Fig. 8:**
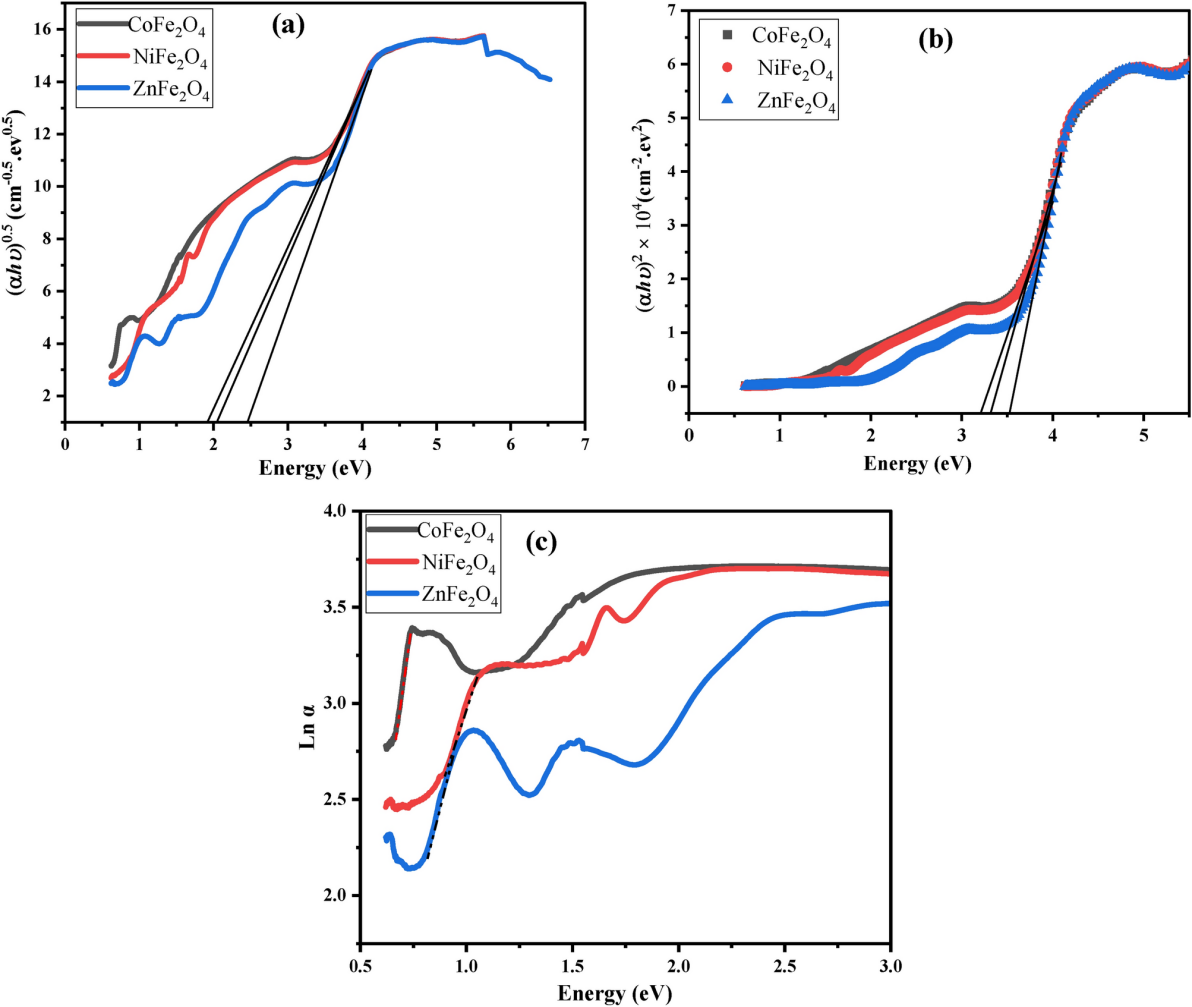
Band Gap and Urbach Energy Analysis. Plots of (αhν) ^0.5^ (**a**), (αhν)^2^ (**b**), and Ln(α) (**c**) as functions of photon energy for CoFe_2_O_4_, NiFe_2_O_4_, and ZnFe_2_O_4_. Results indicate direct and indirect band gap energies (3.3–3.6 eV) and structural disorder levels derived from Urbach energy values.

The extrapolation of the straight line with x-axis gives the direct band gap **(Eg**_**dir.**_**)** and the indirect band gap **(Eg**_**ind.**_**)** as shown in Fig. 8 (a,b). The obtained enrgy gap values are listed in Table 5. From these values, it can be seen that there is only a small variation between the investigated ferrites provide further evidence that the investigated ferrites have almost the same crystal structure. The Urbach energy* E*_*u*_ is a measure of structural disorder in a material and is calculated from the exponential decrease of the absorption coefficient (α) of the material as a function of photon energy ^78^. The Urbach energy can be calculated from α of the material over a range of photon energies. The Urbach energy *E*_*u*_ of the investigated samples can be calculated by fitting an exponential function to the absorption coefficient, and extracting the decay constant Fig. 8 (c). The Urbach energy provides an indication of the stability of the material, with higher Urbach energies corresponding to more sTable materials. This can be useful for the design and optimization of materials for various applications ^79,80^.

6 $$\alpha = {\alpha }_{o} {e}^{\frac{\left(h\nu - {E}_{g}\right)}{{E}_{u}}}$$

The Oxygen vacancies produced in the structure which disturb the system’s band structure and increased the Urbach energy value which listed in Table 5.

The lowest Urbach energy was found in the CoFe_2_O_4_ sample indicating the lowest density of the structure disorder.

The values of α and extinction coefficient (K) are related to the interaction of the ferrites with the incident radiation. Depending on the wavelength and the material’s energy gap Fig. 7(a, b), the absorption coefficient (α) and the extinction coefficient (K) can be calculated using the following formulas ^79,81^:

7 $$\alpha = (2.303 A)/l$$

8 $${\text{K}} = \frac{\alpha \lambda }{{4{\uppi }}}$$

Figure 7 (a, b) shows that absorption (α) and extinction coefficients (K) vary with wavelength. It is clear from the Figures that CoFe_2_O_4_ has the highest absorption efficiency, while ZnFe_2_O_4_ has the lowest absorption efficiency. This demonstrates the wide range of radiation absorption by ferrites, which is an important factor for their application in various fields.

The refractive index n, real and imajenary parts of dielectric constant ε_r_ and ε_i_ were determined for the investigated samples according to the following equations ^82,83^:

9 $$n= \sqrt{\frac{4R}{{\left(R-1\right)}^{2}}- {K}^{2}}- \frac{\left(R+1\right)}{\left(R-1\right)}$$

10 $$\varepsilon_{{\text{r}}} = {\text{ n}}^{{2}} {-}{\text{ k}}^{{2}} ,$$

11 $${\upvarepsilon }_{\text{i}} = 2\text{nk}$$

where R is the reflectance.

**Fig. 9:**
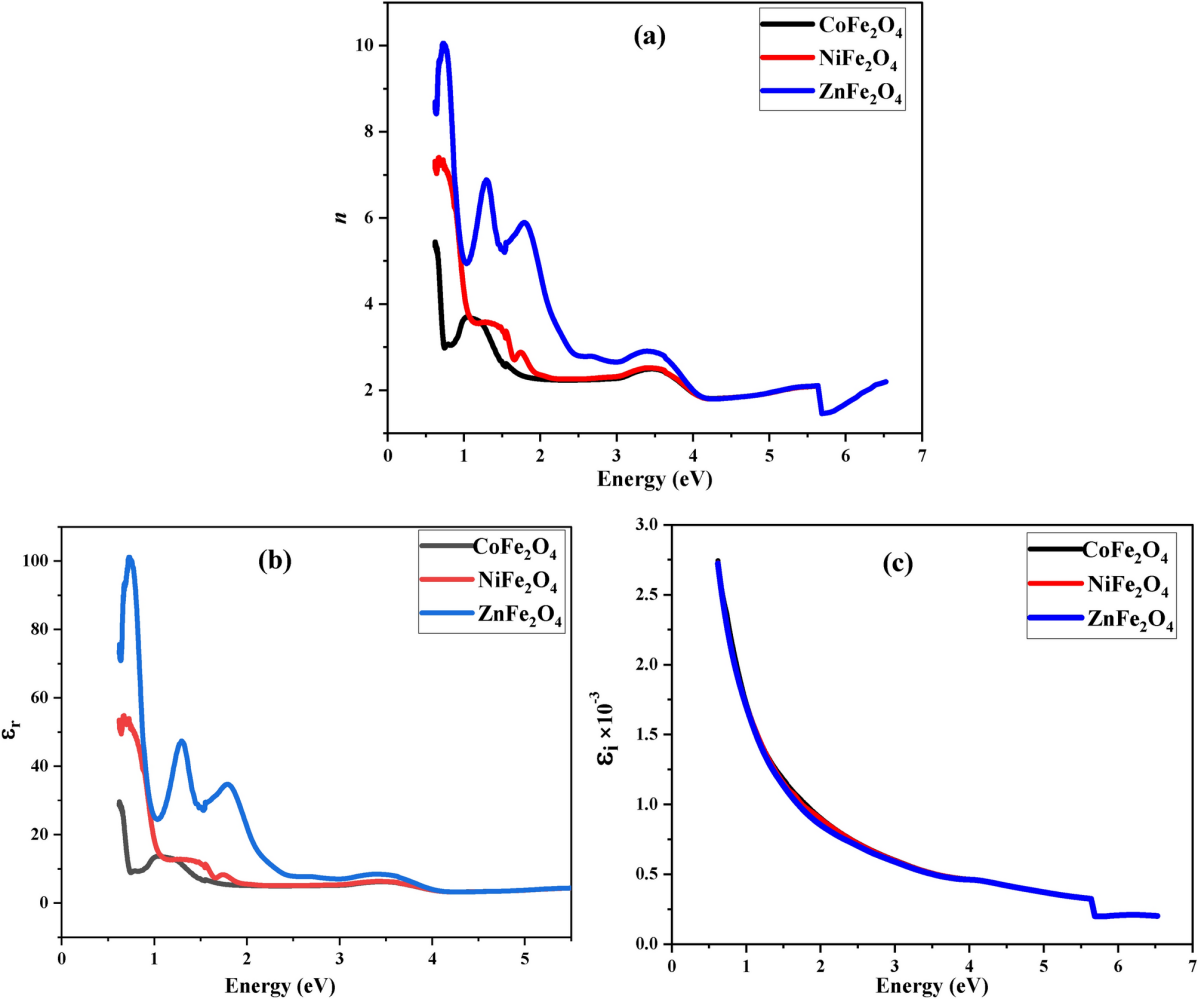
Refractive Index and Dielectric Constants. Variation of refractive index (**a**), real dielectric constant (**b**), and imaginary dielectric constant (**c**) with photon energy for CoFe_2_O_4_, NiFe_2_O_4_, and ZnFe_2_O_4_. CoFe_2_O_4_ shows maximum refractive index values at low photon energy, attributed to oxygen vacancies.

The (Fig. 9 a–c) shows the decay of the refractive index n, ε_r_, and ε_i_ with increasing the incident photon energy. At low energy values, the refractive index ***n*** was maximum due to the presence of the defect levels in the bandgap induced by the oxygen vacancies. These defect levels were responsible for an electronic transition between the valence band and the defect level, which is responsible for the high refractive index at low energy. Additionally, as the incident photon energy increased, the refractive index decreased due to the absence of the defect levels in the bandgap.

Furthermore, the electronic polarization of ions and local field in the optical material are associated to the refractive index. That’s cusing the decreasing of ε_r_ and ε_i_ by increasing the incident photon energy shown in Fig. 9 (b, c).

**Fig. 10:**
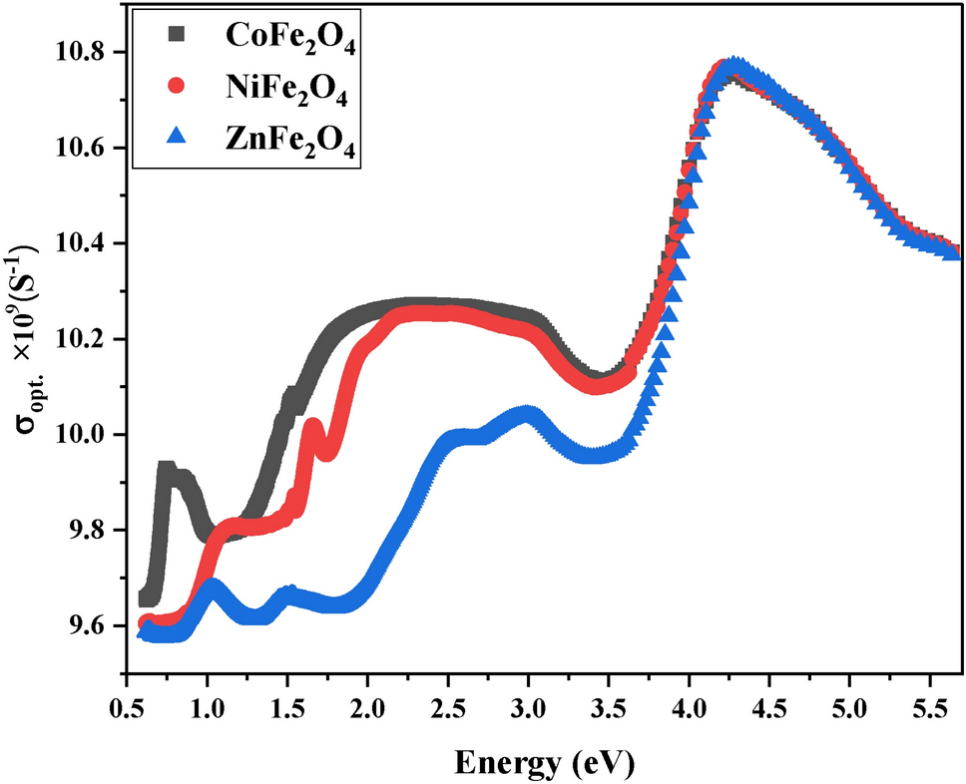
Optical Conductivity. Graph of optical conductivity versus photon energy for CoFe_2_O_4_, NiFe_2_O_4_, and ZnFe_2_O_4_. CoFe_2_O_4_ exhibits the highest conductivity at 4.3 eV, corresponding to electron liberation from valence to conduction bands.

The results of the optical conductivity σ_opt_ measurements show that the σ_opt_ increases with the incident photon energy, reaching a maximum at 4.3 eV. This increasing trend is attributed to the liberation of electrons from the valence band to the conduction band, which is caused by an absorption of the incident photon energy and a decrease in oxygen vacancies. This behavior is demonstrated in Fig. 10. The optical conductivity can be calculated using the following equation ^84,85^:

12 $${\sigma }_{opt.}= \frac{\alpha nc}{4\pi }$$

**Fig. 11:**
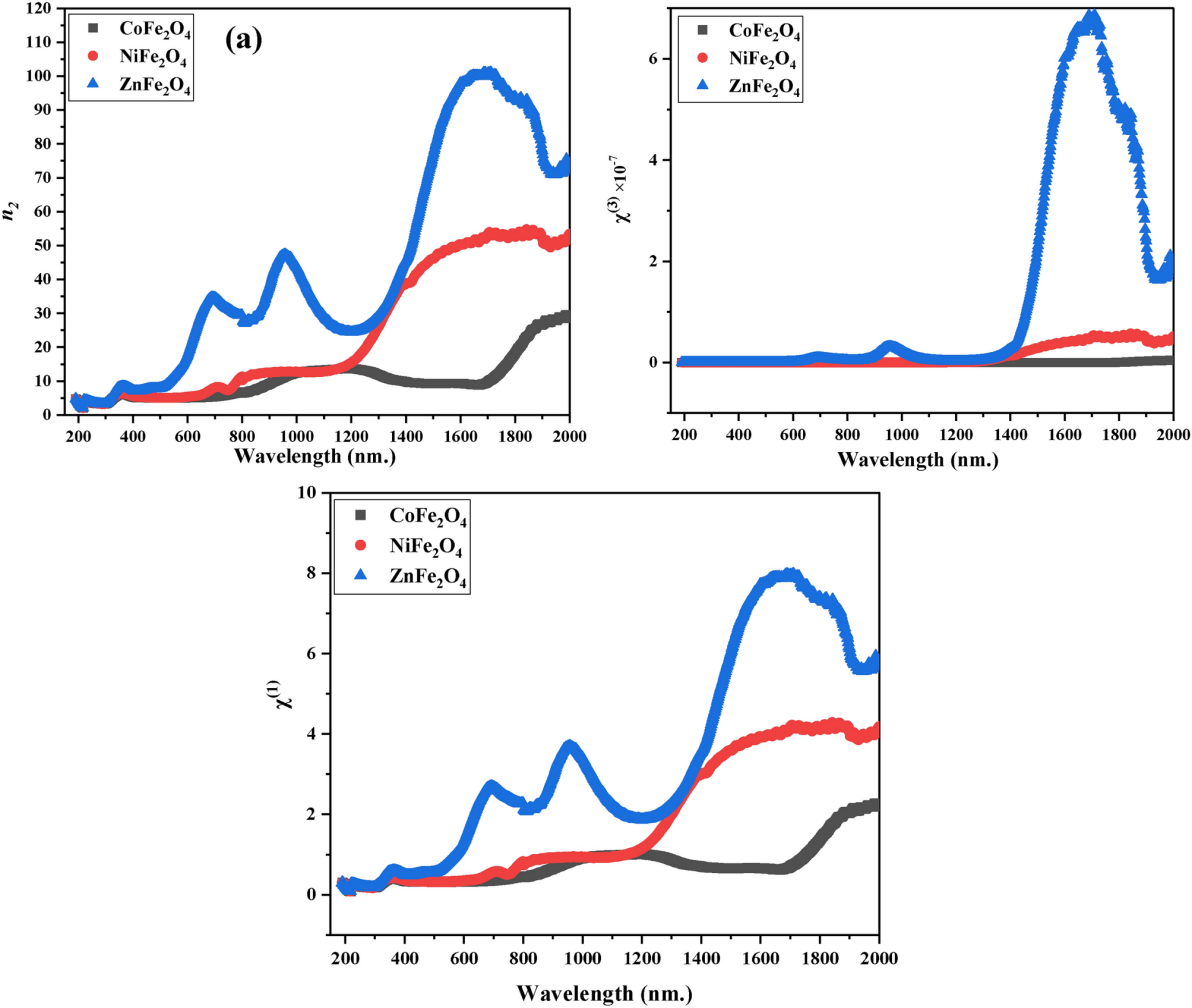
Non-Linear Optical Properties. Plots of non-linear refractive index (**a**), third-order susceptibility (**b**), and linear optical susceptibility (**c**) for CoFe_2_O_4_, NiFe_2_O_4_, and ZnFe_2_O_4_. ZnFe_2_O_4_ displays a peak in short-wave infrared radiation, enhancing its suitability for optoelectronic applications.

The results of this study suggest that ZnFe_2_O_4_ shows an impressive increase in non-linear refractive index (n^2^) and third order of non-linear susceptibility χ^(3)^ in the range of short wave infrared radiation with a peak at 1692 nm. This increase is due to the decrease in mobile charges, which leads to a decrease in material polarization. Additionally, the linear optical susceptibility was also improved. These results Fig. 11 indicate that ZnFe_2_O_4_ is a promising material for non-linear optical χ^(1)^ applications ^83^. The nonlinear parameters such as non-linear refractive index n_2_, 3rd order non-linear optical susceptibility χ^(3)^. Also, the linear optical susceptibility χ^(1)^ was calculated using the following equations.

13 $$P= {\chi }^{(1)}E+ {P}_{NL}$$

14 $${P}_{NL}= {\chi }^{(2)} {E}^{2}+ {\chi }^{(3)}{E}^{3}$$

15 $${\chi }^{(1)}= \frac{({n}^{2}-1)}{4\pi }$$

16 $${\chi }^{(3)}= \frac{\xi }{{\left(4\pi \right)}^{2}} {\left({n}_{o}^{2}-1\right)}^{4}$$

where $$\xi$$ = 1.7 × 10^–10^ (esu).

17 $${n}_{2}= \frac{12\pi {\chi }^{(3)}}{{n}_{o}}$$

### Magnetic properties

**Fig. 12:**
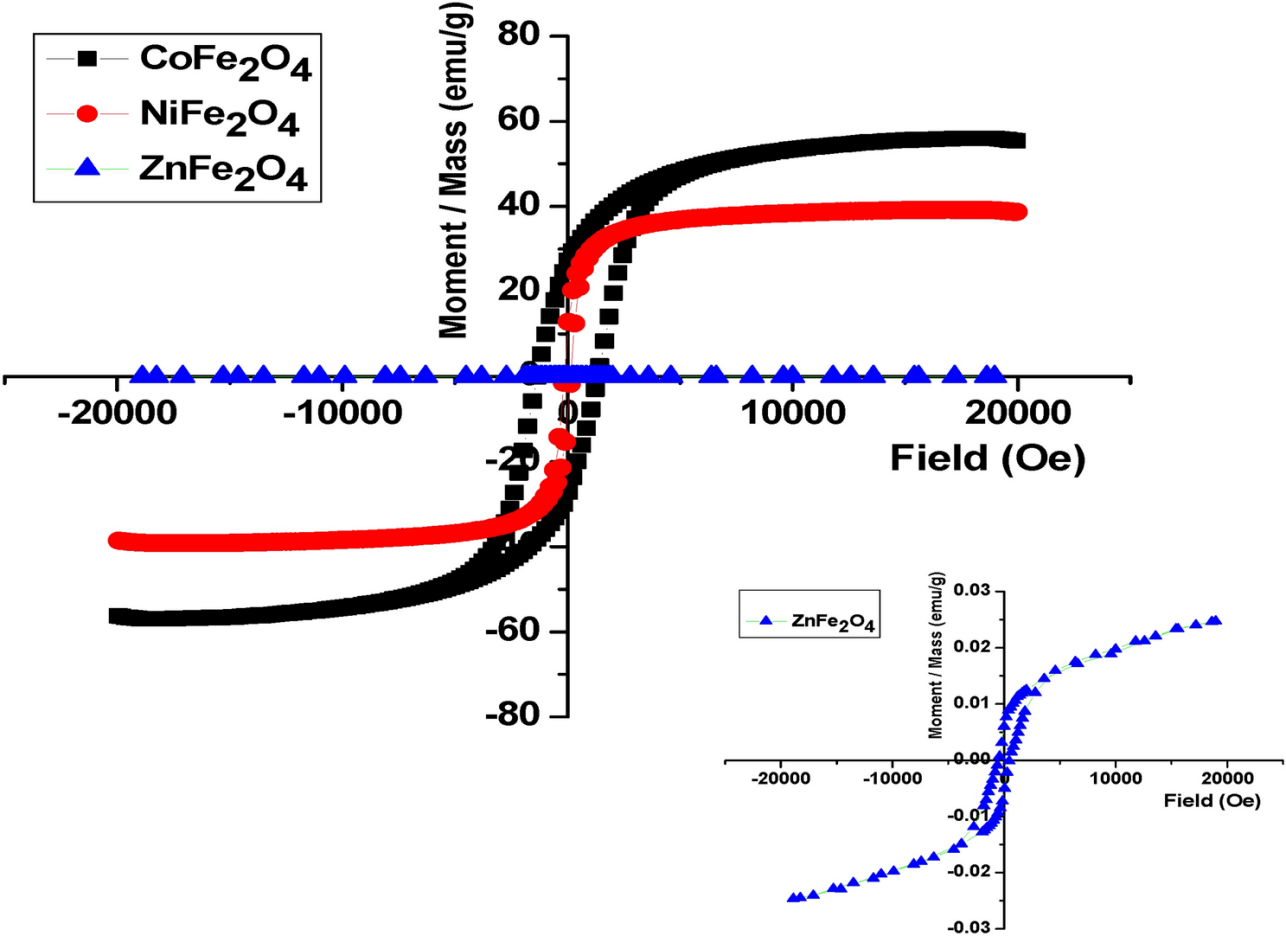
Magnetic Hysteresis Loops. M-H magnetic loops of CoFe_2_O_4_, NiFe_2_O_4_, and ZnFe_2_O_4_ nanoparticles, showing ferrimagnetic behavior for CoFe_2_O_4_ and NiFe_2_O_4_, and antiferromagnetic properties for ZnFe_2_O_4_. Parameters include saturation magnetization, coercivity, and remanent magnetization, emphasizing diverse magnetic properties.

Figure 12 illustrates the M-H magnetic loops of the AFe_2_O_4_ (A: Co, Ni and Zn) spinel ferrites at room temperature. The prepared samples CoFe_2_O_4_ and NiFe_2_O_4_ have ferrimagnetic behavior in the applied field range of -20,000 to 20,000 Oe, while ZnFe_2_O_4_ has antiferromagnetic behavior as shown in the inset of Fig. 12. The values of the saturation magnetization (M_s_), the remanent magnetization (M_r_), the coercivity (H_c_) and the squareness ratio were reported in the Table 6. The magnetic properties of the ferrite materials depends on various parameters such as the morphology, crystallite size, magnetization direction.^34^ It is noted that the magnetization ferrite AFe_2_O_4_ (A: Co, Ni and Zn) are strongly depend on the type of A cation. The highest value of M_s_ was observed for CoFe_2_O_4_ nanoparticles 55.97 emu/g while ZnFe_2_O_4_ has the lowest M_s_ which equals 0.025 emu/g because of the difference in the magnetic moment of A cation. The values of magnetic moment of Fe^3+^, Co^2+^, Ni^2+^ and Zn^2+^ are 5 μB, 3 μB, 2 μB and 0 μB, respectively ^86^. The values of the coercive field are 1355 Oe, 137 Oe and 482 Oe for the nanoferrites CoFe_2_O_4_, NiFe_2_O_4_ and ZnFe_2_O_4_, respectively. The decrease of Hc of the NiFe_2_O_4_ and ZnFe_2_O_4_ than that of the CoFe_2_O_4_ related to the decrease in the anisotropy field and also the domain wall energy.

**Table 6:** The saturation magnetization (M_s_), the remanent magnetization (M_r_), the coercive field (H_c_), and the anisotropy constant (K) for the investigated samples.

Samples	M_s_ (emu/g)	M_r_ (emu/g)	H_c_ (Oe)	SQR = M_r_/M_s_	K (erg/g)
CoFe_2_O_4_	56	27	1355	0.48	79,042
NiFe_2_O_4_	39	5	137	0.13	5595
ZnFe_2_O_4_	25 × 10^–3^	6 × 10^–3^	483	0.24	12.57

Magnetic interaction in the spinel ferrite (AB_2_O_4_) are originated from the three main magnetic interactions:

i. Intrasublattice magnetic interaction (A-A),
ii. Exchange magnetic interactions (B-B),
iii. super exchange magnetic interactions (A-B)

CoFe_2_O_4_ and NiFe_2_O_4_ are inverse spinel where the Co^2+^ and Ni^2+^ ions are placed in the octahedral B site while, Fe^3+^ ions are present in the tetrahedral A and octahedral B sites. Therefore, the magnetic interactions in the Co ferrite are ${Fe}_{A}^{3+}-$ ${Fe}_{B}^{3+}$, ${Fe}_{A}^{3+}- {Co}_{B}^{2+}$, ${Fe}_{A}^{3+}-$ ${Fe}_{A}^{3+}$, ${Co}_{B}^{2+}- {Co}_{B}^{2+}$, and ${Fe}_{B}^{3+}- {Fe}_{B}^{3+}$. While, the magnetic interactions in Ni ferrite are ${Fe}_{A}^{3+}-$ ${Fe}_{B}^{3+}$, ${Fe}_{A}^{3+}- {Ni}_{B}^{2+}$, ${Fe}_{A}^{3+}-$ ${Fe}_{A}^{3+}$, ${Ni}_{B}^{2+}- {Ni}_{B}^{2+}$, and ${Fe}_{B}^{3+}- {Fe}_{B}^{3+}$. The super-exchange magnetic interaction ${Fe}_{A}^{3+}-$ ${Fe}_{B}^{3+}$ is main magnetic interaction and also is the stronger than the other and corresponding to the ferrimagnetic behavior for the samples CoFe_2_O_4_ and NiFe_2_O_4_. The M_s_ of the CoFe_2_O_4_ is greater than that of the NiFe_2_O_4_ owing to the magnetic moment of Co^2+^ ions is higher than that of Ni^2+^ ions. While, ZnFe_2_O_4_ is a normal spinel ferrite where Zn^2+^ ions are placed in the tetrahedral sites [A]. there is no magnetic interaction between A-A cations owing to the non magnetic properties of Zn ions. The only magnetic interactions in ZnFe_2_O_4_ are originated between ${Fe}_{B}^{3+}- {Fe}_{B}^{3+}$ so the saturation magnetization of ZnFe_2_O_4_ is lower than CoFe_2_O_4_ and NiFe_2_O_4_. Also ${Fe}_{B}^{3+}- {Fe}_{B}^{3+}$ interaction is responsible for the antiferromagnetic properties of ZnFe_2_O_4_ ferrite. These results are compatible with the optical results where the exchange interaction responsible for the ferrimagnetic behavior can also influence the optical properties of the ferrite, particularly in the infrared region.

The anisotropy constant (K) value indicates a dipole’s resistance to being destroyed by a reverse field and is strogly depends on thr coerceivity (H_c_) and the saturation magnetization (M_s_) according to the following equation^87^:

18 $$K=\frac{{H}_{C}\times {M}_{S}}{0.96}$$

The values of K were reported in Table 6. The ratio between the M_r_ and M_s_ is define as the squareness ratio R which refres to the interaction between the magnetic domains. It has been reported that when R = 0.5, the coherent rotations occur in randomly oriented, non-interacting particles. While in the investigated nanosamples, the value of R < 0.5 which indicates that the magnetic particles are interacted magnetostatically ^69^.

## Conclusion

Spinel ferrites of the form AFe_2_O_4_ (A: Co, Ni, Zn) were successfully synthesized and thoroughly characterized. Their optical and magnetic properties were systematically investigated.

- The prepared ferrites exhibit high transmittance in the visible, near-infrared (NIR), and short-wave infrared (SWIR) regions, making them suiTable for applications such as:Transparent electrodes in electronic devices (e.g., touch screens, solar cells, displays). Optical and infrared filters for cameras and sensors. Optical waveguides and resonators.
- High nonlinear optical parameters (χ⁽^3^⁾ ≈ 8) were observed in the wavelength range of 1400–1900 nm, highlighting their potential for optoelectronic devices.
- The samples demonstrated excellent UV-blocking capabilities (α = 55 cm⁻^1^ in the 200–300 nm range), making them ideal for UV detectors and photodiodes.
- Band gap energies ranged from 3.3 to 3.6 eV, influencing their absorption and reflection characteristics. Higher energy photons are absorbed, while lower energy photons are reflected.
- Refractive indices were found to increase at low energy values (n ≈ 10 for energies < 1 eV), attributed to higher defect concentrations.
- Optical conductivity increased significantly with higher energy photons (up to 10 × 10⁹ S⁻^1^ at 4.5 eV), indicating their efficiency for light-sensing applications.
- CoFe_2_O_4_ and NiFe_2_O_4_ exhibited ferrimagnetic behavior, while ZnFe_2_O_4_ displayed antiferromagnetic properties. These magnetic characteristics influence light-matter interactions, making them promising for advanced technological applications.

The combination of tunable optical and magnetic properties positions AFe_2_O_4_ spinel ferrites as versatile materials for a wide range of applications, including optoelectronics, photonics, and magnetic devices. Their unique properties make them an exciting area for further research and development.

## Data Availability

Derived data supporting the findings of this study are available from the corresponding author on request.
